# Treatments for recurrent hepatocellular carcinoma: laparoscopic or open repeat liver resection, how to make a decision?

**DOI:** 10.1007/s13304-023-01503-w

**Published:** 2023-04-20

**Authors:** Chenhao Zhang, Jinli Zheng, Li Jiang

**Affiliations:** 1 Department of Breast Surgery, Affiliated Hospital of Southwest Medical University, Luzhou, 64600 China; 2 Department of Liver Surgery, West China Hospital of Sichuan University, Chengdu, Sichuan Province China; 3 Department of Liver Transplantation Center, West China Hospital of Sichuan University, Chengdu, Sichuan China

Dear Editor

Recently, we read the paper titled “Laparoscopic versus open repeat liver resection for recurrent hepatocellular carcinoma in hepatectomy patients: inverse probability of treatment weighting” reported by Eun Sung Jeong et al. published in Updates in Surgery [1]. Laparoscopic repeat liver resection (LRLR) and open repeat liver resection (ORLR) in the treatment of recurrent hepatocellular carcinoma (HCC) were compared, and they found that patients in the LRLR group achieved better short-term outcomes than those in the ORLR group. Although their findings seem convincing and interesting, a few questions may deserve further discussion.

Certainly, LRLR could shorten the postoperative hospital stay, and this study proposed that patients who underwent LRLR could obtain better short-term oncologic outcomes [1]. This was an interesting phenomenon in the clinic, because in our subconscious, the short-term outcomes were comparable in these two treatments. However, the sample was relatively small in this study. In our opinion, the primary tumor pathological features in the first operation also make sense, which may affect the short-term outcomes in the situation of tumor recurrence. In our center, we evaluated recurrent tumors combined with the primary tumor features at the first operation, such as the size of the tumor, the number of tumors, and the MVI status of the pathology. What we mentioned above was the independent risk factors for HCC. [2–6] Thus, we should take the first operation situation into consideration before making a decision on recurrent HCC. In this study, the number of patients who received laparoscopic liver resection (LLR) in the LRLR group was higher than that in the ORLR group at the first operation (64.0% VS 32.0%, *p* = 0.024). The tumor situation at first operation may be significantly different for these patients, because the tumor size or location would affect the method of hepatectomy [6, 7].

Generally, another important risk factor for recurrent HCC is the time of tumor recurrence. In previous studies, the long-term outcomes of tumor recurrence within 1 year after hepatectomy were worse than those of tumor recurrence beyond 1 year after liver resection [8]. Therefore, it was necessary to compare the time of tumor recurrence when we analyzed the different treatments for recurrent HCC. On the other hand, in this study, the location of the tumor recurrence was different, which might present a different message for the tumor. The prevalent hypothesis was multicentric occurrence (MO) and intrahepatic metastasis (IM). [8] MO indicates de-novo carcinogenesis and IM indicates primary cancer relapse. The different tumor recurrence types would result in different outcomes. IM indicated a tumor with more aggression, resulting in a high risk of recurrence, although the patient had undergone resection again. Thus, the authors should add the comparison of the pathological diagnose in first operation and second operation for these patients, respectively, to identify the tumor origins, if possible, we could perform the genetic test to clarify the tumor origins. In summary, although LRLR could obtain a better short-term outcome in this study, we could not ignore other risk factors, especially the tumor status at the first operation and the pathological diagnosis in these patients. Therefore, we should take the first operation situation into consideration when we make a decision on recurrent HCC.

## Data Availability

This study is a letter for the editor, no data.
